# Association Among Self‐Reported Bruxism, Stress and Headache in a Sample of Adolescents: A Cross‐Sectional Study

**DOI:** 10.1111/joor.70255

**Published:** 2026-07-21

**Authors:** Amanda Carolina Flor Soares, Daniella Andrade Guimarães, Janice Simpson de Paula, Ana Paula Varela Brown Martins

**Affiliations:** ^1^ Federal University of Juiz de Fora Campus Governador Valadares Gov. Valadares Minas Gerais Brazil; ^2^ Department of Dentistry Federal University of Minas Gerais Belo Horizonte Minas Gerais Brazil; ^3^ Department of Dentistry Federal University of Santa Catarins Florianópolis Santa Catarina Brazil

**Keywords:** adolescent, habits, prevalence, primary headache disorders, psychological stress

## Abstract

**Objective:**

This study evaluated the prevalence of self‐reported awake bruxism and self‐reported sleep bruxism among elementary and middle school students and its association with stress and primary headache.

**Methods:**

Three questionnaires were applied: questions about parafunction and behavioural habits for bruxism diagnosis, International Headache Society questionnaire for headache prevalence analysis, Lipp's inventory symptoms of stress (ISSL) for stress assessment. For the analysis of the data, the chi‐square test was performed to compare proportions with 95% CI and the relative risk ratio test was also applied to study association with age and race. The level of significance was 5%.

**Results:**

A total of 209 adolescents participated in the study. The prevalence was 3.8% for self‐reported sleep bruxism, 8.13% for self‐reported awake bruxism and 49.3% for bruxism, regardless of the frequency. There was an association among self‐reported sleep bruxism, headache (*p* = 0.037) and stress (*p* = 0.000); self‐reported awake bruxism, age (*p* = 0.024) and stress (*p* = 0.007); as well as similarly for the simultaneity of self‐reported awake bruxism and sleep bruxism.

**Conclusion:**

There was also an association among stress, some symptoms of sleep bruxism and parafunctional and occupational habits. The findings suggest a significant association between self‐reported sleep bruxism, self‐reported awake bruxism, stress and self‐reported headache.

## Graphical abstract

This graphical abstract illustrates the conceptual pathway investigated in this cross‐sectional study: stress experienced by adolescents may be associated with self‐reported bruxism (sleep and awake), which in turn shows a significant association with primary headache (tension‐type or migraine). The findings indicate significant associations among self‐reported sleep bruxism, self‐reported awake bruxism, stress and headache in adolescents, suggesting that these conditions may be interconnected within a broader biopsychosocial framework.

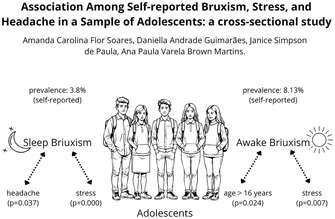

## 1 Introduction

During adolescence, young people are exposed to a host of novel interpersonal stressors that may confer risk for other psycho‐emotional conditions, if poorly managed [1]. For example, stress may be associated with oral behaviours, such as bruxism, by inducing motor responses to the masticatory muscle [2].

Bruxism is currently conceptualized as a behaviour manifested as repetitive jaw‐muscle activity characterized by clenching or grinding of the teeth and/or by bracing or thrusting of the mandible, in healthy individuals [3, 4]. Different behaviours are observed during sleep—sleep bruxism (SB) and wakefulness—awake bruxism (AB) [4, 5]. Studies investigating the prevalence of bruxism across diverse age groups and genders have yielded inconsistent results [6, 7].

The aetiology of bruxism is considered multifactorial and is not yet fully understood; however, several factors are believed to contribute to its development [8]. Among psychological factors, an association between bruxism and elevated stress levels has been consistently reported [9]. Bruxism has been linked to periods of heightened psychological stress, during which individuals may discharge accumulated tension through repetitive jaw‐muscle activity. It has also been associated with social discomfort, states of deep concentration and emotional responses such as anger [10]. Indeed, psychological stress is frequently reported among individuals with bruxism and is considered a potential contributing factor to its onset and perpetuation [11]. Notably, higher stress scores have been significantly associated with higher self‐reported awake bruxism scores [12].

Bruxism can present with diverse clinical manifestations and may occur at any age [13]. The clinical presentation is heterogeneous and may vary considerably among individuals. Signs may include teeth grinding, abnormal tooth wear, tongue indentation, masticatory muscle hypertrophy and jaw locking, whereas symptoms may encompass morning headache, facial pain or fatigue and reports of tooth grinding or jaw‐clenching sounds during sleep—whether self‐reported or noted by a bed partner [14]. The extent of damage to stomatognathic structures depends on the balance—or imbalance—between the muscular forces exerted during bruxism activity and the resistance of the target structures, including the teeth, muscles, and temporomandibular joints (TMJs) [15]. Regardless of age, potential consequences include abnormal tooth wear or fracture, tension‐type headache, masticatory muscle pain or fatigue, temporomandibular disorders and wear and/or fracture of dental restorations [16]. As such, bruxism may interfere with an adolescent's quality of life, particularly in domains related to functional limitations and social well‐being, including family dynamics [17].

Given that bruxism, headache and stress can all cause pain and/or discomfort, many adolescents frequently miss school due to pain‐related complaints [18]. The relationship between bruxism and headache has been documented in the literature [19]. Headaches are common during childhood and become even more prevalent during adolescence [20]. Primary headaches can lead to significant disability in adolescents, adversely affecting academic performance, relationships with family and peers and overall well‐being [21]. However, the available literature on this topic remains limited; therefore, additional data may contribute to a more comprehensive understanding of the potential associations among bruxism, stress and tension‐type headache or migraine in adolescent populations.

Adolescence is a critical developmental stage marked by heightened emotional reactivity and increased exposure to social and environmental stressors. In contexts of socioeconomic vulnerability, these stressors may be further intensified, potentially shaping stress‐related behaviours. Given that bruxism is currently conceptualized as a behaviour influenced by central and psychosocial mechanisms, examining its association with stress‐related factors during this developmental period may yield clinically and epidemiologically relevant insights.

Although the association between bruxism and headache has been previously documented in the literature, the novelty of the present study lies in its specific focus on the adolescent population. The simultaneous evaluation of three interrelated conditions—bruxism, stress and headache—within a socioeconomically vulnerable adolescent sample adds epidemiological value to the existing literature, contributing to a more comprehensive understanding of their potential comorbid relationship during this critical developmental window. This research aimed to estimate the prevalence and frequency of self‐reported bruxism among adolescents, as well as to evaluate the associations among self‐reported bruxism, psychological stress and headache. The null hypotheses of this study were: (1) there is no association between bruxism and stress; and (2) there is no association between bruxism and headache. The conceptual hypothesis is that these three clinical conditions are associated, possibly in a comorbid relationship with one another.

## 2 Methods

This cross‐sectional study was approved by the Research Ethics Committee of the Federal University of Juiz de Fora (Juiz de Fora, Minas Gerais, Brazil—protocol number 2.518.080) and was conducted in accordance with the ethical standards of the Declaration of Helsinki. The study design and reporting follow the STROBE (Strengthening the Reporting of Observational Studies in Epidemiology) guidelines, ensuring methodological transparency, clarity and reproducibility (Vandenbroucke et al. [22]). The STROBE checklist is given in Data S1. All participants aged 16 years or older provided written informed consent prior to inclusion. For participants under 16 years of age, parental or guardian consent was required, and the consent form was sent home for signature. Data collection took place between March and July 2018.

Data were collected through face‐to‐face interviews conducted by trained interviewers, who explained the aims of the study to all potential participants. Participants were selected based on availability and willingness to participate; no randomization tool was applied, as a convenience sampling strategy was intentionally adopted given the face‐to‐face recruitment approach conducted within the school environment. The inclusion criteria were: being between 13 and 19 years of age and in generally good health, with no restrictions regarding race or social group; being regularly enrolled in and attending a public school in Governador Valadares (Minas Gerais, Brazil)—specifically students from the ninth grade of middle school to the third year of high school, in both morning and afternoon shifts; and being able to complete the questionnaire independently. Participants were excluded if they presented with systemic diseases that could confound the results (rheumatoid arthritis, polyarthritis, heart disease or diabetes mellitus), facial paralysis, facial trauma, or were using medications that could interfere with the results (analgesics, anti‐inflammatory drugs, antidepressants or anticonvulsants). The required sample size was estimated for a cross‐sectional prevalence study, assuming a 95% confidence level, a 5% margin of error, and an expected proportion of 50%. The target population consisted of 333 students, yielding a minimum sample of 179 participants after finite population correction. To account for potential non‐response, an additional 10%–20% was considered (≈199–224 participants). Of the 333 enrolled students, 209 met the eligibility criteria and agreed to participate.

Efforts were made to minimize potential sources of bias throughout the study design and data collection process. The study was conducted in a single public school selected by convenience; however, within this setting, all eligible students were invited to participate in order to minimize selection bias. To reduce information bias, data were collected using structured questionnaires, and all members of the research team received standardized training to ensure consistency in administration. Given the cross‐sectional nature of the study, reverse causality cannot be excluded, as exposure and outcome were measured simultaneously. Therefore, all findings are interpreted as associations rather than causal relationships. For analytical purposes, age was dichotomized at 16 years, corresponding approximately to the sample mean and to a transitional phase between middle and late adolescence, to explore potential developmental differences within the sample.

### 2.1 Self‐Reported Bruxism Assessment

The prevalence and frequency of self‐reported bruxism were assessed using a structured questionnaire. For sleep bruxism (SB), participants responded to the following questions: ‘Do you grind or clench your teeth while sleeping?’ and ‘Did anyone ever tell you that you grind your teeth in your sleep?’ Responses were recorded on a 5‐point frequency scale: never, less than 1 day/night per month, 1–3 days/nights per month, 1–3 days/nights per week and 4 or more days/nights per week. A participant was classified as having self‐reported SB if they responded affirmatively to either question with a frequency of at least 1–3 nights per week, corresponding to high frequency. For awake bruxism (AB), participants responded to the questions ‘Do you clench or touch your teeth together?’ and ‘Do you hold, tighten or tense your muscles without chewing or touching your teeth together?’ using a different 5‐point scale: never, a brief period of the time, some of the time, most of the time and all of the time. A participant was classified as having self‐reported AB if they responded affirmatively to either question with a frequency of at least ‘most of the time,’ corresponding to high frequency [23].

### 2.2 Stress Assessment

Stress was evaluated using the Lipp Stress Symptom Inventory (LSSI), a validated instrument in which participants marked an ‘X’ next to symptoms experienced in the last 24 h, the last week and the last month [24].

### 2.3 Headache Assessment

Self‐reported headache was assessed using questions from a validated questionnaire [25], including: ‘How long have you had headaches?’ and ‘What is the type of pain?’ (e.g., throbbing, pressure, stabbing or burning), as well as questions regarding pain duration and frequency.

### 2.4 Statistics

Data were analysed using the chi‐square test to compare proportions between groups. For the dependent variables—self‐reported sleep bruxism, self‐reported awake bruxism and both combined—odds ratios and their respective 95% confidence intervals were calculated. The level of statistical significance was set at 5%. All analyses were performed using SPSS (Statistical Package for Social Sciences for Windows), version 23.0.

## 3 Results

Of the 333 enrolled students, 209 participated in the study (23.4% middle school and 76.6% high school), with ages ranging from 13 to 19 years (mean ± standard deviation: 15.83 ± 1.28 years). The remaining 124 students were excluded due to not meeting the inclusion criteria, declining to participate or providing incomplete responses to the questionnaires (Figure 1). The frequency distribution of self‐reported SB, self‐reported AB, stress and headache is presented in Table 1.

**FIGURE 1:**
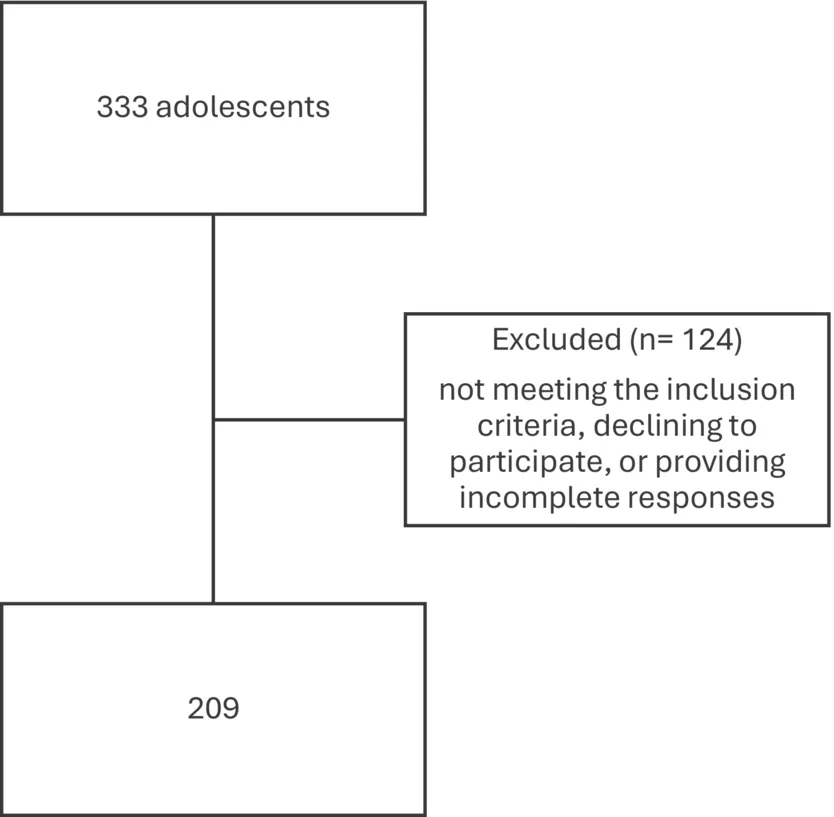
Flow diagram of the participants' selection.

**TABLE 1:** The descriptive data of the adolescent population (*n* = 209).

Variables	*N* (%)
Frequency of self‐reported SB	8 (3.8%)
Grinding or Clenching teeth (*n* = 209)	
Never	190 (90.9%)
Sometimes	12 (5.7%)
Frequent	7 (3.3%)
Third‐party reports of teeth grinding or clenching (*n* = 209)	
Never	192 (91.9%)
Sometimes	11 (5.3%)
Frequent	5 (2.4%)
Frequency of self‐reported AB	17 (8.13%)
Clenching or touching teeth together (*n* = 202)^a^	
Never	102 (48.85%)
Sometimes	87 (41.6%)
Frequent	13 (6.25%)
Holding, bracing or thrusting of the mandible (*n* = 207)^b^	
Never	133 (63.6%)
Sometimes	63 (30.1%)
Frequent	11 (5.3%)
Placing tongue between teeth (*n* = 204)^c^	
Never	119 (56.9%)
Sometimes	64 (30.6%)
Frequent	21 (10.0%)
Biting nails (*n* = 202)^a^	
Never	78 (37.3%)
Sometimes	68 (32.5%)
Frequent	56 (26.8%)
Holding between teeth or biting objects (*n* = 205)^d^	
Never	93 (44.5%)
Sometimes	80 (38.3%)
Frequent	32 (15.3%)
Headache (*n* = 199)^e^	
No headache	29 (18.7%)
Pulsating quality	85 (40.7%)
Pressing or tightening (non‐pulsating) quality	29 (13.9%)
Others	56 (26.8%)

^a^ 7 adolescents did not answer the first question about self‐reported awake bruxism.

^b^ 2 adolescents did not answer the second question about self‐reported awake bruxism.

^c^ 5 adolescents did not answer about self‐reported oral behaviour (Placing tongue between teeth).

^d^ 4 adolescents did not answer about self‐reported oral behaviour (Holding between teeth or biting objects).

^e^ 10 adolescents did not answer about self‐reported headaches.

Regarding the frequency of self‐reported bruxism, 3.8% of participants reported SB and 8.13% reported AB. A total of 103 adolescents (49.3%) reported both SB and AB across different frequency levels. Among oral parafunctional habits, nail biting was the most frequently reported behaviour, with 32.5% of participants reporting it sometimes and 26.8% reporting it frequently.

According to the LSSI, stress was present in 49.3% of the adolescents. With respect to headache characteristics, 85 participants (40.7%) reported pulsating pain quality, 29 (13.9%) reported pressing pain quality and 56 (26.8%) reported other pain qualities. Regarding headache frequency, 16.7% reported experiencing headache on fewer than 15 days per month (between 10 and 14 days) and 16.3% reported an average headache duration of 1 day, followed by 14.4% who reported durations of 4–12 h.

Regarding the associations between self‐reported SB and headache characteristics and stress, statistically significant associations were found between self‐reported SB and pulsating headache quality [OR = 3.34 (95% CI: 1.45–7.66); *p* = 0.004], and between self‐reported SB and stress [OR = 3.88 (95% CI: 2.17–6.96); *p* < 0.001] (Table 2).

**TABLE 2:** Association between self‐reported sleep bruxism and headache pain characteristics and stress in adolescents from public schools in Governador Valadares, Minas Gerais, Brazil (*n* = 202).

Variables	Categories	Total	Self‐reported sleep bruxism		OR (95% CI)	*p*
			No *n* (%)	Yes *n* (%)		
Age	≤ 16	137	71 (51.8%)	66 (48.2%)	Ref.	
	> 16	65	31 (47.7%)	34 (52.3%)	1.18 (0.66–2.13)	0.345
Headache	No report	37	26 (70.3%)	11 (28.7%)	Ref.	
	Pulsing quality	82	34 (41.5%)	48 (58.5%)	3.34 (1.45–7.66)	0.004
	Pressing quality	27	14 (51.9%)	13 (48.1%)	2.20 (0.78–6.17)	0.136
	Others	56	28 (50.0%)	28 (50.0%)	2.36 (0.98–5.69)	0.055
Stress	No	104	69 (66.3%)	35 (33.7%)	Ref.	
	Yes	98	33 (33.7%)	65 (66.3%)	3.88 (2.17–6.96)	0.000

*Note:* Missing data: *n* = 7.

Abbreviation: OR, Odds Ratio (Confidential Interval 95%).

When comparing self‐reported AB with headache characteristics, age and stress, significant associations were observed between self‐reported AB and age older than the sample mean (16 years) [OR = 1.97 (95% CI: 1.05–3.71); *p* = 0.024], pulsating headache quality [OR = 2.42 (95% CI: 1.11–5.31); *p* = 0.027] and stress [OR = 2.14 (95% CI: 1.20–3.81); *p* = 0.007] (Table 3).

**TABLE 3:** Association between self‐reported awake bruxism and headache pain characteristics and stress in adolescents from public schools in Governador Valadares, Minas Gerais, Brazil (*n* = 201).

Variables	Categories	Total	Self‐reported awake bruxism		OR (95% CI)	*p*
			No *n* (%)	Yes *n* (%)		
Age	≤ 16	136	61 (44.9%)	75 (55.1%)	Ref.	
	> 16	65	19 (29.2%)	46 (70.8%)	1.97 (1.05–3.71)	0.024
Headache	No report	39	21 (53.8%)	18 (46.2%)	Ref.	
	Pulsing quality	80	26 (32.5%)	54 (67.5%)	2.42 (1.11–5.31)	0.027
	Pressing quality	27	10 (37.0%)	17 (63.0%)	1.98 (0.73–5.41)	0.181
	Others	55	23 (41.8%)	32 (58.2%)	1.62 (0.71–3.71)	0.251
Stress	No	103	50 (48.5%)	53 (51.5%)	Ref.	0.007
	Yes	98	30 (30.6%)	68 (69.4%)	3.88 (2.17–6.96)	

*Note:* Missing data: *n* = 8.

Abbreviation: OR, Odds Ratio (Confidential Interval 95%).

When examining the associations between self‐reported SB and AB combined and the study variables, significant associations were found with age older than the sample mean (16 years) [OR = 1.96 (95% CI: 1.07–3.57); *p* = 0.020], pulsating headache quality [OR = 2.21 (95% CI: 1.10–4.83); *p* = 0.047] and stress [OR = 1.98 (95% CI: 1.13–3.46); *p* = 0.012] (Table 4).

**TABLE 4:** Association between the combination of self‐reported sleep bruxism and self‐reported awake bruxism and headache pain characteristics and stress in adolescents from public schools in Governador Valadares, Minas Gerais, Brazil (*n* = 202).

Variables	Categories	Total	Self‐reported sleep bruxism and self‐reported awake bruxism		OR (95% CI)	*p*
			No *n* (%)	Yes *n* (%)		
Age	≤ 16	136	74 (54.4%)	62 (45.6%)	Ref.	
	> 16	66	25 (37.9%)	41 (62.1%)	1.96 (1.07–3.57)	0.020
Headache	No report	39	24 (61.5%)	15 (38.5%)	Ref.	
	Pulsing quality	81	34 (42.0%)	47 (58.0%)	2.21 (1.01–4.83)	0.047
	Pressing quality	27	11 (40.7%)	16 (59.3%)	2.33 (0.85–6.34)	0.099
	Others	55	30 (54.5%)	25 (45.5%)	1.33 (0.58–3.07)	0.500
Stress	No	103	59 (57.3%)	44 (42.7%)	Ref.	0.012
	Yes	99	40 (40.4%)	59 (59.6%)	1.98 (1.13–3.46)	

*Note:* Missing data: *n* = 7.

Abbreviation: OR, Odds Ratio (Confidential Interval 95%).

With the exception of nail biting, a positive association was observed between the frequency of awake bruxism‐related symptoms—including facial pain (*p* < 0.001), masticatory muscle fatigue (*p* < 0.001), morning temporal headache (*p* = 0.001), jaw locking or sticking (*p* = 0.004) and tooth or gum sensitivity (*p* < 0.001)—parafunctional and occupational habits, and the frequency of self‐reported stress among adolescents (Table 5).

**TABLE 5:** Association of symptoms of self‐reported sleep bruxism and parafunctional and occupational habits during wakefulness with stress in adolescents from public schools in Governador Valadares, Minas Gerais, Brazil.

Morning bruxism symptoms and awake parafunctional and occupational habits				Stress		
				No	Yes	*p*
Variables		Frequency	Total	*n* (%)	*n* (%)	
Upon Awakening	Facial Pain	None	183	104 (56.8%)	79 (43.2%)	0.000
		Low	17	2 (11.8%)	15 (88.2%)	
		High	8	0 (0.0%)	8 (100.0%)	
	Tiredness of masticatory muscles	None	160	95 (59.4%)	65 (40.6%)	0.000
		Low	27	7 (25.9%)	20 (74.1%)	
		High	19	2 (10.5%)	17 (89.5%)	
	Morning temporal headache	None	129	78 (60.5%)	51 (39.5%)	0.001
		Low	59	24 (40.7%)	35 (59.3%)	
		High	19	4 (21.1%)	15 (78.9%)	
	Locking or Sticking Jaw	None	190	103 (54.2%)	87 (45.8%)	0.004
		Low	10	3 (30.0%)	7 (70.0%)	
		High	8	0 (0.0%)	8 (100.0%)	
	Teeth or Gum Sensitivity	None	115	74 (64.3%)	41 (35.7%)	0.000
		Low	58	22 (37.9%)	36 (62.1%)	
		High	30	8 (26.7%)	22 (73.3%)	
Awake	Putting Tongue Between Teeth	None	119	71 (59.7%)	48 (40.3%)	0.007
		Low	64	23 (35.9%)	41 (64.1%)	
		High	21	9 (42.9%)	12 (57.1%)	
	Biting Nails	None	78	37 (47.4%)	41 (52.6%)	0.549
		Low	68	38 (55.9%)	30 (44.1%)	
		High	56	27 (48.2%)	29 (51.8%)	
	Biting or Holding Objects Between Teeth	None	93	62 (66.7%)	31 (33.3%)	0.000
		Low	80	37 (46.3%)	43 (53.8%)	
		High	32	7 (21.9%)	25 (78.1%)	

## 4 Discussion

This cross‐sectional study found that a small proportion of adolescents reported a high frequency of self‐reported SB and AB, with self‐reported AB being more commonly reported. Notably, a considerable number of adolescents reported experiencing bruxism regardless of frequency. The null hypotheses were rejected, as significant associations were observed between self‐reported bruxism and both stress and headache. Specifically, significant associations were found between self‐reported SB and stress and pulsating headache quality, as well as between self‐reported AB and stress. Furthermore, when self‐reported SB and AB were considered together, associations with stress and older age were also identified.

The present study found a prevalence of 3.8% for self‐reported SB, 8.13% for self‐reported AB, and 49.3% when frequency was not considered. These estimates are within the ranges reported in previous studies on adolescents, which have described prevalence rates of 5%–52.9% for SB and 4.1%–34.5% for AB [5, 6, 7, 26, 27]. Approximately 7.3% of adolescents have been reported to experience both AB and SB [26]. In the present study, only higher frequencies of self‐reported bruxism episodes—defined as at least 1–3 nights per week—were considered, as sporadic episodes are unlikely to carry clinical significance [28]. When applying this threshold, prevalence estimates were lower; however, this approach may better reflect clinically relevant bruxism and its potential consequences, particularly when other risk factors are present [4].

The prevalence of AB and SB observed in the present study is consistent with previous reports, including the absence of significant gender differences [5, 7, 29, 30]. However, the association between bruxism prevalence and sex remains uncertain in the literature, with some studies reporting more severe bruxism among women [10, 31, 32] and others indicating a higher likelihood of this behaviour among men [33].

As in adults, the aetiology of SB in adolescents is not fully understood, but several factors have been implicated, including genetics, environmental factors, stress, anxiety, upper airway obstruction, sleep structure, sleep habits, sleep‐disordered breathing, self‐reported poor sleep quality, attention deficit hyperactivity disorder, neurological disorders, medications, video game addiction, childhood trauma and gastroesophageal reflux [8, 29, 30, 34, 35, 36, 37, 38]. The relationship between stress, emotional tension and bruxism—particularly during wakefulness—has been previously highlighted by Manfredini et al. [39], in which ‘stress sensitivity,’ defined as an abnormal reactivity to stressors, was identified as one of the main factors differentiating individuals with bruxism from those without. Individuals with bruxism exhibited increased susceptibility to stress, and it was proposed that bruxism may reflect a reduced ability to cope effectively with emotional load, possibly representing a maladaptive stress response. Increased stress sensitivity in adolescents could facilitate the translation of emotional tension into sustained jaw muscle activity, suggesting that bruxism may function as a maladaptive coping mechanism. Individuals with bruxism are twice as likely to report severe stress [40], and adolescents with bruxism have been found to report higher levels of perceived stress [29]. Additionally, stress may be associated with alterations in sleep quality and depth, which could in turn be related to SB [26].

The present study found significant associations between stress and self‐reported SB (*p* < 0.001), self‐reported AB (*p* = 0.007) and the combination of both (*p* = 0.012). Stress, defined as the perception of exceeding one's resources to meet demands [41], is prevalent during adolescence due to the physical and social changes characteristic of this developmental period [42], which may negatively impact behaviour and lifestyle [43]. Stressors that are particularly prevalent during adolescence [33, 34, 35]—such as bullying [33, 34, 35, 44, 45], neighbourhood violence [34, 46] and family instability [29, 35, 47]—may increase the risk for negative secondary outcomes [36, 48] and could be associated with school dropout [37, 38, 49, 50]. In this regard, stress has been linked to several cognitive impairments, including slower processing speed, impaired executive function and memory deficits [39, 51]. The significant association between self‐reported AB and perceived stress observed in this study is consistent with previous research [5, 26, 29]. AB is thought to be closely related to psychological factors and psychopathological symptoms, including stress [40, 52]. In a study conducted with Chinese adolescents, childhood trauma and perceived stress scores were identified as risk factors for bruxism, and perceived stress levels were found to mediate the relationship between childhood trauma and bruxism onset [29]. The complex relationship between bruxism and psychosocial factors may also influence self‐report, as responses might reflect psychological distress rather than actual masticatory muscle behaviour [4]. Emotional states may increase muscle tension and activity, potentially triggering or aggravating bruxism, even during sleep or wakefulness. Furthermore, anxiety and stress may interfere with sleep onset and overall sleep quality [32]. Regarding SB specifically, alterations in sleep duration and quality have been associated with an increased incidence of bruxism [4, 53]. Sleep disturbances may negatively affect emotional health, leading to general discomfort, irritability, stress and anxiety, possibly due to elevated cortisol levels [4]. These mechanisms may help explain the associations observed in the present study.

Sex‐related factors may influence psychological vulnerability, coping strategies and responses to psychosocial stressors, with some evidence suggesting greater vulnerability among females [54]. Several factors may contribute to this vulnerability, including biological influences such as hormonal fluctuations [55], psychological factors related to coping mechanisms [56], and sociocultural influences such as societal expectations that may place additional pressure on women [57]. Although these factors may partly explain the higher prevalence of emotional disorders and, consequently, bruxism among women, the present study did not observe significant sex‐related differences in stress levels or in self‐reported SB or AB. This finding may be related to the age of the participants, as these factors may be more pronounced among adult women than among adolescents.

The distinction between SB and AB is relevant when examining associations with psychosocial factors. Although AB appears to be closely related to psychosocial variables, the aetiology of SB is considered more complex, involving arousal‐related mechanisms, sleep fragmentation and potential interactions with systemic medical conditions [58]. From this perspective, SB should not be interpreted solely as stress‐related behaviour, but rather as a multifactorial motor activity occurring within a broader neurophysiological framework [58]. It has also been suggested that rhythmic masticatory muscle activity (RMMA) may provide relief from psychological pressure and that bruxism may represent a behavioural outlet when mental overload occurs [59]. Within this framework, the associations observed in the present study should be interpreted cautiously, acknowledging that psychological stress may represent one component within a more complex biopsychosocial model.

This study also found significant associations between morning bruxism‐related symptoms—including facial pain, masticatory muscle fatigue, headache, jaw stiffness and gum sensitivity—and stress (*p* < 0.001). Similar findings have been reported in adolescents aged 11–19 years [60]. Facial pain has been associated with bruxism and may be exacerbated by stress, potentially creating a self‐reinforcing cycle [26]. Adolescents experiencing pain are more likely to be absent from school and report higher stress levels [18]. Pain has also been closely linked to sleep disturbances, which may further increase stress and compromise school attendance [61]. These findings highlight the importance of identifying and managing pain‐related conditions in adolescent populations.

A significant association was also found between self‐reported SB and pulsating headache quality, as well as between morning temporal headache and stress (*p* < 0.001). Morning temporal headache was common in this sample, and its frequency showed a positive association with the prevalence of stress among participants. These findings are consistent with previous evidence indicating that transient morning jaw‐muscle pain or fatigue associated with self‐reported SB demonstrates moderate but significant agreement with polysomnographic diagnosis [23], supporting the hypothesis that SB frequency may be related to the occurrence of morning headaches [62]. Although these associations suggest a possible interrelationship among SB, headache, and stress, the underlying pathophysiological mechanisms remain incompletely understood.

An association between self‐reported SB and headache symptoms was observed in the present study. Previous research has shown that adolescents experience high rates of primary headaches, including 11% for migraine and 17% for tension‐type headache [20]. Patients with headache have also reported greater dental contact, masticatory tension, stress and facial pain [63]. In adolescent populations, headaches have been associated with poor sleep habits, lower physical activity levels, skipping breakfast, caffeine overuse and smoking [64]. Sleep characteristics appear to represent a potential link between SB and headache.

The association between self‐reported AB and headache has been previously reported [18]; however, this association was not observed in the present study. This discrepancy may be attributed to methodological differences. In the present study, headache was analysed as a general category rather than being differentiated into migraine or tension‐type headache. Only pain characteristics—such as pulsating or pressing quality—were considered. This methodological approach aimed to minimize potential misclassification, as distinguishing between headache subtypes would require a more specific diagnostic framework beyond the scope of self‐reported questionnaires. Therefore, the association observed between self‐reported SB and headache should be interpreted within the context of general headache symptoms.

Recent literature has highlighted the role of mandibular bracing—characterized by sustained, low‐level muscle contraction occurring regardless of tooth contact and largely outside conscious awareness, particularly during wakefulness—as a relevant behavioural expression of bruxism [65]. This tonic muscle activity may represent a pathway through which emotional load is translated into prolonged muscle tension. Self‐reported bracing has been associated with psychological traits such as anxiety and depression, supporting the hypothesis that emotional distress may contribute to sustained jaw muscle tension [65]. Within this framework, the association observed in the present study between stress, self‐reported AB and tension‐type headache appears clinically coherent. Prolonged low‐level muscle contraction may be associated with muscle fatigue and increased stiffness, potentially contributing to temporal headaches. Although causal inferences cannot be drawn due to the cross‐sectional design, these findings are consistent with the perspective that bracing behaviours may represent a behavioural link between psychosocial distress and musculoskeletal symptoms.

The directionality of the associations observed in the present study cannot be established due to its cross‐sectional design, and bidirectional relationships among stress, headache, and bruxism behaviours should be considered. Stress is frequently reported by individuals with migraine as a trigger for headache episodes [66]. Acute stress may induce biological changes that lower the threshold for migraine attacks, while repeated exposure to behavioural or physiological stressors may contribute to dysregulated allostatic responses. Allostatic load, defined as the cumulative physiological burden resulting from chronic stress [65], may alter the normal functioning of neurobiological systems and contribute to functional and structural changes in brain networks [67, 68]. These alterations may increase vulnerability to both headache and stress‐related behaviours, such as bruxism. Consequently, migraine, stress and psychological symptoms may interact within a self‐reinforcing cycle in which each condition may contribute to the persistence or exacerbation of the others, ultimately affecting quality of life [69]. Within this framework, the associations observed in the present study may reflect shared underlying mechanisms rather than a direct causal relationship among these conditions.

This study has several limitations that should be considered when interpreting the findings. First, the identification of self‐reported bruxism, stress and headache through questionnaires may introduce potential biases and affect the accuracy of responses. However, this approach is consistent with methodologies commonly employed in previous research [19], allowing for comparability of findings across different populations. While self‐report enables large‐scale recruitment and individual‐level screening, it does not allow quantification of the intensity or duration of masticatory muscle activity [70], and responses may be influenced by psychological distress, personal beliefs [71], cognitive awareness and memory recall [72]. Second, bruxism was assessed through self‐report rather than a standardized diagnostic instrument. Although the STAB (Standardized Tool for the Assessment of Bruxism) represents a more rigorous assessment approach, it was not available at the time of data collection. Notably, the validated Brazilian Portuguese version of the STAB was published in September 2025, postdating both the data collection phase and the initial submission of this manuscript. Future studies should consider incorporating this instrument to allow for more standardized and comparable bruxism assessments in adolescent populations. Third, the use of a convenience sampling strategy, rather than a probabilistic or randomized approach, limits the generalizability of the findings to the broader adolescent population. Future studies should consider employing randomized sampling methods to enhance the representativeness of the sample and the external validity of the results. Fourth, muscle and neuromuscular disorders were not explicitly included in the exclusion criteria, which could act as potential confounders when interpreting self‐reported masticatory muscle symptoms, including fatigue, pain or stiffness. Fifth, the study was conducted in a single public school, which may further limit the generalizability of the findings and introduce socioeconomic bias. Sixth, the limited availability of scientific evidence on the associations among these variables in adolescents makes direct comparisons with the existing literature difficult. The diversity of questionnaires and methodologies across studies also hinders direct comparisons. Finally, restricting the analyses to univariate models represents an additional limitation, as confounding factors were not controlled. Therefore, the reported odds ratios should be interpreted with caution.

The findings of this study demonstrate that self‐reported AB and SB are present among adolescents and that, with respect to age, self‐reported AB was more commonly reported among adolescents older than the sample mean of 16 years. The associations shared by self‐reported SB and AB include psychological stress and older age (> 16 years), while self‐reported headache was associated with self‐reported SB and stress. Taken together, these findings suggest that both self‐reported SB and AB among adolescents may be associated with stress, and that self‐reported headache may be associated with self‐reported SB and stress. The simultaneous occurrence of self‐reported bruxism, headache and stress in adolescents highlights associations that may warrant attention from a multidisciplinary perspective. While cross‐sectional design does not allow causal inferences, these findings suggest that these conditions may coexist and potentially be associated with aspects of adolescents' quality of life, including academic performance and school attendance.

## Author Contributions

**Amanda Carolina Flor Soares:** investigation, writing – original draft, data curation, conceptualization. **Janice Simpson de Paula:** conceptualization, methodology, writing – review and editing, writing – original draft, formal analysis. **Daniella Andrade Guimarães:** conceptualization, data curation, investigation, resources, writing – original draft. **Ana Paula Varela Brown Martins:** conceptualization, methodology, supervision, project administration, writing – original draft, writing – review and editing.

## Funding

The authors have nothing to report.

## Conflicts of Interest

The authors declare no conflicts of interest.

## Supporting information


**Data S1:** STROBE Statement—Checklist of items that should be included in reports of cross‐sectional studies.

## Data Availability

The data that support the findings of this study are available from the corresponding author upon reasonable request.
